# Psychometric Performance of a Substance Use Symptom Checklist to Help Clinicians Assess Substance Use Disorder in Primary Care

**DOI:** 10.1001/jamanetworkopen.2023.16283

**Published:** 2023-05-26

**Authors:** Theresa E. Matson, Kevin A. Hallgren, Gwen T. Lapham, Malia Oliver, Xiaoming Wang, Emily C. Williams, Katharine A. Bradley

**Affiliations:** 1 Kaiser Permanente Washington Health Research Institute, Seattle; 2 Department of Health Systems and Population Health, University of Washington School of Public Health, Seattle; 3 Health Services Research & Development Center for Innovation for Veteran-Centered and Value-Driven Care, Veterans Affairs Puget Sound Health Care System, Seattle, Washington; 4 Department of Psychiatry and Behavioral Sciences, University of Washington School of Medicine, Seattle; 5 Center for the Clinical Trials Network, National Institute on Drug Abuse, National Institutes of Health, Bethesda, Maryland; 6 Department of Medicine, University of Washington School of Medicine, Seattle

## Abstract

**Importance:**

Substance use disorders (SUDs) are underrecognized in primary care, where structured clinical interviews are often infeasible. A brief, standardized substance use symptom checklist could help clinicians assess SUD.

**Objective:**

To evaluate the psychometric properties of the Substance Use Symptom Checklist (hereafter symptom checklist) used in primary care among patients reporting daily cannabis use and/or other drug use as part of population-based screening and assessment.

**Design, Setting, and Participants:**

This cross-sectional study was conducted among adult primary care patients who completed the symptom checklist during routine care between March 1, 2015, and March 1, 2020, at an integrated health care system. Data analysis was conducted from June 1, 2021, to May 1, 2022.

**Main Outcomes and Measures:**

The symptom checklist included 11 items corresponding to SUD criteria in the *Diagnostic and Statistical Manual for Mental Disorders* (Fifth Edition) (*DSM-5*). Item response theory (IRT) analyses tested whether the symptom checklist was unidimensional and reflected a continuum of SUD severity and evaluated item characteristics (discrimination and severity). Differential item functioning analyses examined whether the symptom checklist performed similarly across age, sex, race, and ethnicity. Analyses were stratified by cannabis and/or other drug use.

**Results:**

A total of 23 304 screens were included (mean [SD] age, 38.2 [5.6] years; 12 554 [53.9%] male patients; 17 439 [78.8%] White patients; 20 393 [87.5%] non-Hispanic patients). Overall, 16 140 patients reported daily cannabis use only, 4791 patients reported other drug use only, and 2373 patients reported both daily cannabis and other drug use. Among patients with daily cannabis use only, other drug use only, or both daily cannabis and other drug use, 4242 (26.3%), 1446 (30.2%), and 1229 (51.8%), respectively, endorsed 2 or more items on the symptom checklist, consistent with *DSM-5* SUD. For all cannabis and drug subsamples, IRT models supported the unidimensionality of the symptom checklist, and all items discriminated between higher and lower levels of SUD severity. Differential item functioning was observed for some items across sociodemographic subgroups but did not result in meaningful change (<1 point difference) in the overall score (0-11).

**Conclusions and Relevance:**

In this cross-sectional study, a symptom checklist, administered to primary care patients who reported daily cannabis and/or other drug use during routine screening, discriminated SUD severity as expected and performed well across subgroups. Findings support the clinical utility of the symptom checklist for standardized and more complete SUD symptom assessment to help clinicians make diagnostic and treatment decisions in primary care.

## Key Points

**Question:**

What are the psychometric properties of a Substance Use Symptom Checklist used routinely in primary care among patients reporting high-risk cannabis and/or other drug use?

**Findings:**

In this cross-sectional study of 23 304 positive screens for daily cannabis or any other drug use, the 11-item checklist provided scaled, unidimensional information on the presence and severity of substance use disorder. The checklist performed well across patient age, sex, race, and ethnicity.

**Meaning:**

The findings of this study support the use of the checklist in primary care as a tool to aid clinicians in eliciting patient symptoms, identifying a spectrum of substance use disorder severity, and clinical decision-making based on diagnostic criteria.

## Teaser

This cross-sectional study evaluates the psychometric properties of the Substance Use Symptom Checklist to assess substance use disorder in a primary care setting among adult patients reporting daily cannabis and/or other drug use.

## Introduction

More than 55 million adults in the United States use cannabis or other drugs, with cannabis being most common.^1^ The health and addictive risks vary by substance but generally increase with the frequency and quantity of use.^2^ Overall, 7% of US adults meet diagnostic criteria for substance use disorder (SUD),^3^ defined as having 2 or more of 11 criteria by the *Diagnostic and Statistical Manual of Mental Disorders *(Fifth Edition) (*DSM-5*).^4^ However, the proportion of patients who receive an SUD diagnosis is much lower (0.8%-4.6%).^5,6,7,8,9^ Low rates of diagnosis decrease opportunities for patients to receive treatment^10,11^ despite evidence-based options (eg, pharmacotherapy for opioid use disorder, behavioral treatments for cannabis and stimulant use disorders).^12,13,14^

Brief, validated substance use screens, recommended in primary care by the US Preventive Services Task Force,^15^ typically ask about frequency of cannabis and other drug use and are useful for screening for SUDs. While these brief screens have been validated for SUDs generally,^16^ they do not provide information on *DSM-5* SUD symptoms. Clinicians need to assess consequences or symptoms resulting from substance use to identify the presence and severity of an SUD. However, clinicians may lack time and training to do so consistently. Brief, standardized assessments offer clinicians a way to assess SUDs and are preferable in general medical settings to full diagnostic interviews,^17^ which are time-consuming (10-45 minutes) and have specific training and supervision requirements.^18^ Brief, standardized assessments balance patient and clinician burden while offering a way to help clinicians diagnose SUDs and assess patient treatment needs.^19,20^ While brief SUD assessments have been validated in research and specialty settings,^21,22^ their performance when used in general medical settings, with responses documented in electronic health records (EHRs), has not been psychometrically evaluated.

As part of behavioral health integration, Kaiser Permanente Washington began routinely screening patients for past-year cannabis and other drug use followed by Substance Use Symptom Checklist assessment when patients reported daily cannabis or any other drug use on screens.^5,23,24,25,26,27,28^ This effort provided a unique opportunity to evaluate the clinical application of the Substance Use Symptom Checklist using data collected as part of routine care. Although clinically informative and practical for use in primary care to engage patients in discussions of problems they are experiencing due to cannabis or other drug use,^5,27^ its psychometric performance as a tool to assess SUD severity is untested. The objective of this study was to evaluate the psychometric properties of the Substance Use Symptom Checklist (ie, symptom checklist) when used routinely among primary care patients reporting daily cannabis use, other drug use, or both to support its use in general medical settings.

## Methods

### Design, Data, and Setting

This cross-sectional study used EHR data from Kaiser Permanente Washington. Kaiser Permanente Washington is a large integrated health care system providing primary care across 32 clinics in Washington state. This study received approval, waivers of consent, and HIPAA authorization to use existing EHR data from the Kaiser Permanente Washington Health Research Institute institutional review board. Researchers followed the Strengthening the Reporting of Observational Studies in Epidemiology (STROBE) checklist.

#### Behavioral Health Screening Procedures

Beginning in 2015, adult patients (≥18 years) were asked to complete a 7-item behavioral health screen (on paper or via the patient EHR portal) (eFigure 1 in Supplement 1) as part of routine care, prompted annually with EHR reminders. The screen included separate questions for cannabis (“How often in the past year have you used marijuana?”) and other drug use (“How often in the past year have you used an illegal drug (not marijuana) or used a prescription medication for non-medical reasons?”).^28^ For both cannabis and other drug use questions, response options were: “never,” “less than monthly,” “monthly,” “weekly,” and “daily or almost daily” use.^29^ Staff entered screen results into the EHR prior to clinician visits. More than 90% of primary care patients completed annual cannabis and/or other drug screening.

#### Substance Use Symptom Checklist Procedures

The EHR prompted staff to ask patients to complete the symptom checklist on paper when patients reported cannabis use “daily or almost daily” or any drug use (ie, response other than “never”) and enter responses into the EHR. The decision to use “daily or almost daily” as the threshold for a positive cannabis screen came directly from the health system—based on who might be most likely to have an SUD as well as health system capacity to respond to positive screens—and is consistent with a validation study demonstrating that a threshold of daily cannabis use had high specificity.^30^

The symptom checklist (eFigure 2 in Supplement 1) was based on 11 *DSM-5* criteria^4^ to elicit SUD symptoms in primary care. It was developed in partnership with frontline clinicians and implemented with behavioral health screening as a tool to facilitate conversations between clinicians and their patients who reported high-risk substance use.^5^ Approximately 73% to 78% of patients who reported high-risk substance use completed the symptom checklist.^28^

### Sample

**Figure 1.**
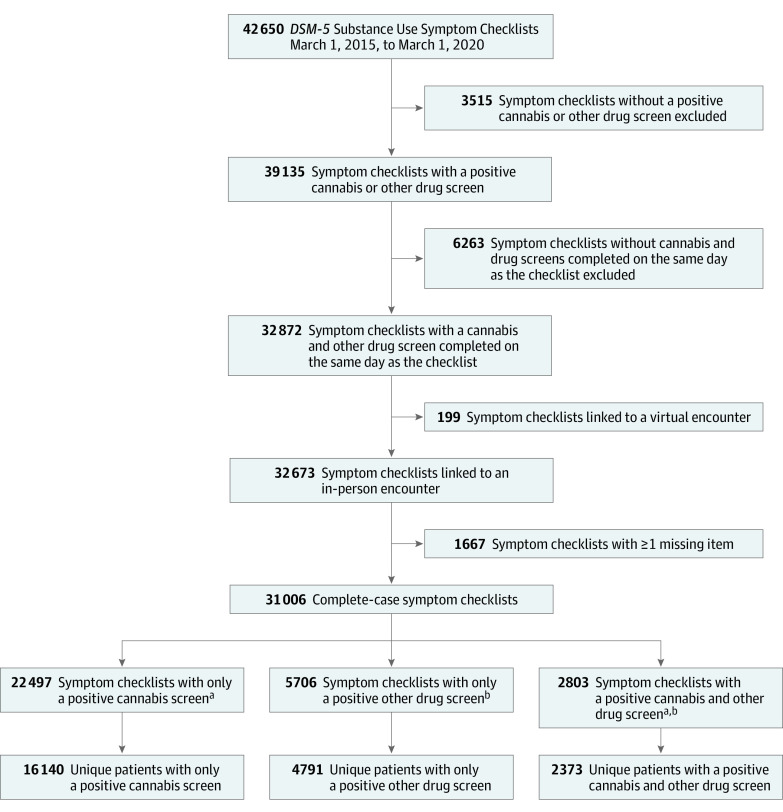
Process of Selecting *Diagnostic and Statistical Manual of Mental Disorders* (Fifth Edition) (*DSM-5*) Substance Use Symptom Checklists for Inclusion in Analyses *DSM-5* Substance Use Symptom Checklists are typically administered after a positive cannabis and/or other drug use screen, as part of routine primary care. Positive cannabis and/or other drug use screens were used to define subsamples in which to test the psychometric properties of the checklist. A single, random checklist for each subsample was selected. ^a^Cannabis screens were considered positive if a patient indicated daily or almost daily cannabis use in the past year. ^b^Other drug screens were considered positive if a patient indicated any other drug use in the past year.

Adult patients (≥18 years) were included if they had at least 1 primary care visit from March 1, 2015, to March 1, 2020, and completed 1 or more eligible symptom checklists. Symptom checklists (N = 42 650) were eligible if prompted by daily cannabis use and/or any other drug use screens (39 135 [91.8%]) and completed on the same day as screening (32 872 [84.0%]). We excluded incomplete symptom checklists (1667 [5.1%]) and those linked to a virtual encounter due to differences in workflow (199 [0.6%]). Each symptom checklist fell into 1 of 3 mutually exclusive subsamples, those triggered by (1) daily cannabis use only (n = 22 497); (2) other drug use only (n = 5706); or (3) both daily cannabis and other drug use (n = 2803). However, patients could have completed more than 1 symptom checklist over the study period and thereby be included in more than 1 symptom checklist subsample or in 1 subsample multiple times. Within each symptom checklist subsample, we randomly selected 1 symptom checklist per patient (Figure 1).

### Measures

#### Substance Use Symptom Checklist

Patients self-reported the presence or absence of 11 *DSM-5* symptom criteria on the symptom checklist. The symptom checklist had a past-year timeframe, consistent with diagnostic standards.^4^ The symptom checklist was scored by summing symptom criteria counts (0-11).^31^ *DSM-5* considers 2 to 3 criteria mild; 4 to 5, moderate; and 6 to 11, severe SUD.

#### Demographic Characteristics

Demographic characteristics collected from patients and documented in the EHR by the health system were used to approximate patients’ lived identities and experiences. This included age (18-24, 25-44, 45-64, and ≥65 years), sex (female or male) listed on legal forms of identification, self-identified race (American Indian or Alaska Native, Asian, Black, Native Hawaiian or Pacific Islander, or White), and ethnicity (Hispanic or non-Hispanic).^32^ Participants reporting other or unknown race or unknown ethnicity were included in descriptive tables and full sample analyses but excluded from race- and ethnicity-stratified analyses.

### Statistical Analysis

Patient characteristics were described across the 3 symptom checklist subsamples: daily cannabis use only, other drug use only, and both daily cannabis and other drug use. Psychometric analyses of the symptom checklist were conducted using item response theory (IRT) in each of the symptom checklist subsamples. IRT aims to model the relationships that an observable set of measures (eg, SUD criteria reported on symptom checklists) have with an unobservable or latent trait (eg, SUD severity).^33^ A 2-parameter logistic IRT model using maximum likelihood was fit with the *mirt* package in R version 4.2.3 (R Project for Statistical Computing) (eAppendix 7 in Supplement 1).^34^ We performed 3 IRT analyses, 1 for each symptom checklist subsample.

#### Unidimensionality

IRT assumes that the latent variable (SUD severity) exists along a unidimensional continuum. This unidimensionality is consistent with current diagnosing standards characterizing SUD as a single brain disorder with varying severity.^4,31,35^ Using a confirmatory approach, we examined the fit of unidimensional IRT models by comparing model fit indices (range, 0-1) to standard cutoffs for acceptable fit: comparative fit index (CFI) greater than 0.95, root mean square error of approximation (RMSEA) less than 0.05, and standardized root mean square residual (SRMSR) less than 0.05.^36,37^ Standardized factor loadings (λ) characterized the relationship between each item and latent SUD severity^38^; loadings (range, −1 to 1) with absolute value of 0.40 or greater were considered substantive.^39^ We examined residual correlation between items after fitting the unidimensional model to identify potential item clusters violating the IRT assumption of local independence and may indicate an unmodeled second factor. If correlations were small (absolute values <0.10),^40^ a unidimensional model was considered appropriate.^40,41^

#### Item Characteristics

IRT models used 2 parameters, discrimination (*a*) and severity (*b*) to characterize the association between how patients responded to items on the symptom checklist and their latent SUD severity. Discrimination characterized how well an item differentiated patients with higher vs lower SUD severity; values could range from −∞ to ∞ (but typically range from 0 to 2)^42^ with higher values indicative of better discrimination. Severity (also known as difficulty) characterized the value along the continuum of latent SUD severity at which an item best discriminated, which was also the level of latent SUD severity needed for patients to have a 50% chance of endorsing the item; values could range from −∞ to ∞ (but typically range from −3 to 3), with higher values endorsed at higher levels of SUD severity.^41^ IRT parameters were plotted graphically as item characteristic curves (with discrimination and severity corresponding to an item’s slope and location, respectively) to illustrate how the probability of endorsing each of the 11 items (y-axis) was associated with one’s latent SUD severity (x-axis).^41,42^

#### Differential Item Functioning and Expected Clinical Impact

Differential item functioning (DIF) could occur if the probabilities of endorsing specific symptom checklist items were influenced by a patient’s broader lived experiences rather than their latent level of SUD severity.^42,43^ A high degree of DIF could be clinically problematic, suggesting, for example, that some items measured SUD differently for different demographic subgroups. For each demographic subgroup (age, sex, race, and ethnicity) within each symptom checklist subsample, we used a likelihood ratio test to statistically test for DIF by comparing a more complex IRT model in which item parameters could vary by demographic subgroup to a simpler model that constrained item parameters to be the same for subgroups (eAppendix 2 in Supplement 1).^43,44^

However, DIF could be present without meaningfully changing clinical interpretation of scores.^43^ Because SUD severity was determined by the number of SUD criteria (0-11), knowing whether DIF led to differences in criteria count may be more clinically meaningful than knowing whether DIF was present for individual items.^44^ We examined differences in the total expected number of criteria (0-11) endorsed by patients with the same level of latent SUD severity to understand whether the test as a whole performed differently across demographic subgroups. We also examined differences in CFIs between models with and without correction for DIF for each demographic subgroup (eAppendix 2 in Supplement 1).^45^

## Results

### Descriptive

A total of 23 304 screens were included (mean [SD] age, 38.2 [5.6] years; 12 554 [53.9%] male patients; 17 439 [78.8%] White patients; 20 393 [87.5%] non-Hispanic patients). Symptom checklist subsamples included 16 140 patients who reported daily cannabis use only, 4791 patients who reported other drug use only, and 2373 patients who reported both daily cannabis and other drug use (Figure 1). All 3 subsamples were predominantly male (8386 [52.0%]; 2693 [56.2%]; and 1475 [62.2%]), aged 25 to 44 years (7576 [46.9%]; 2470 [51.6%]; and 1257 [53.0%]), non-Hispanic (14 252 [88.3%]; 4113 [85.9%]; and 2028 [85.5%]), and White (12 397 [76.8%]; 3391 [70.8%]; and 1734 [73.1%]) (Table 1).

**Table 1.** Sample Characteristics Among Primary Care Patients Who Reported Daily Cannabis Use Only, Other Drug Use Only, and Both Daily Cannabis and Other Drug Use on Routine Screening From March 1, 2015, to March 1, 2020

Characteristic	Patients, No. (%)		
	Daily cannabis use only (n = 16 140)	Other drug use only (n = 4791)	Daily cannabis and other drug use (n = 2373)
Age group, y			
18-24	2813 (17.4)	1245 (26.0)	787 (33.2)
25-44	7576 (46.9)	2470 (51.6)	1257 (53.0)
45-64	4150 (25.7)	842 (17.6)	273 (11.5)
≥65	1601 (9.9)	233 (4.9)	55 (2.3)
Sex			
Female	7754 (48.0)	2097 (43.8)	897 (37.8)
Male	8386 (52.0)	2693 (56.2)	1475 (62.2)
Race			
American Indian or Alaska Native	397 (2.5)	99 (2.1)	60 (2.5)
Asian	544 (3.4)	425 (8.9)	116 (4.9)
Black or African American	1145 (7.1)	286 (6.0)	177 (7.5)
Native Hawaiian or Pacific Islander	247 (1.5)	92 (1.9)	41 (1.7)
White	12 397 (76.8)	3391 (70.8)	1734 (73.1)
Other or unknown	1400 (8.7)	497 (10.4)	244 (10.3)
Ethnicity			
Hispanic	1045 (6.5)	390 (8.1)	195 (8.2)
Not Hispanic	14 252 (88.3)	4113 (85.9)	2028 (85.5)
Unknown	843 (5.2)	287 (6.0)	149 (6.3)

### Prevalence of SUD Criteria

Among patients with daily cannabis use only, other drug use only, or both daily cannabis and other drug use, 4242 (26.3%), 1446 (30.2%), and 1229 (51.8%), respectively, reported symptoms consistent with SUD (Table 2).^4^ For patients with daily cannabis use only, prevalence of each SUD criterion varied from 4.4% (705 patients) for the item neglect roles to 21.9% (3536 patients) for the item tolerance. For patients with other drug use only, they ranged from 11.9% (570 patients) for the item hazardous use to 25.2% (1207 patients) for the item physical or psychological problems. For patients with daily cannabis use and other drug use, they ranged from 18.7% (444 patients) for the item neglect roles to 41.5% (985 patients) for the item tolerance (Table 2).

**Table 2.** Prevalence of SUD Criteria Assessed on the Substance Use Symptom Checklist Among Primary Care Patients Who Reported Daily Cannabis Use Only, Other Drug Use Only, and Both Daily Cannabis and Other Drug Use on Routine Screening From March 1, 2015, to March 1, 2020

Substance Use Symptom Checklist item	Patients, No. (%)		
	Daily cannabis use only (n = 16 140)	Other drug use only (n = 4791)	Daily cannabis and other drug use (n = 2373)
Tolerance	3536 (21.9)	944 (19.7)	985 (41.5)
Withdrawal	1248 (7.7)	760 (15.9)	495 (20.9)
Larger/longer	1501 (9.3)	984 (20.5)	622 (26.2)
Quit/control	1662 (10.3)	828 (17.3)	565 (23.8)
Time spent	774 (4.8)	666 (13.9)	483 (20.4)
Physical or psychological problems	3139 (19.4)	1207 (25.2)	899 (37.9)
Neglect roles	705 (4.4)	728 (15.2)	444 (18.7)
Hazardous use	919 (5.7)	570 (11.9)	494 (20.8)
Social or interpersonal problems	1571 (9.7)	1009 (21.1)	651 (27.4)
Craving	2693 (16.7)	989 (20.6)	876 (36.9)
Activities given up	1043 (6.5)	726 (15.2)	493 (20.8)
Total *DSM-5* SUD criteria			
0-1 Criteria, no SUD	11 898 (73.7)	3345 (69.8)	1144 (48.2)
2-3 Criteria, mild SUD	2585 (16.0)	468 (9.8)	478 (20.1)
4-5 Criteria, moderate SUD	934 (5.8)	237 (4.9)	252 (10.6)
≥6 Criteria, severe SUD	723 (4.5)	741 (15.5)	499 (21.0)

Abbreviations: *DSM-5*, *Diagnostic and Statistical Manual of Mental Disorders* (Fifth Edition); SUD, substance use disorder.

### Unidimensionality

Unidimensional factor models demonstrated excellent fit to the symptom checklist’s 11 items (CFI >0.95; RMSEA <0.05; SRMSR <0.05) (Table 3), indicating they measured latent SUD severity along a unidimensional continuum. Standardized factor loadings ranged from 0.64 to 0.96 (Table 3), and residual correlations were small (absolute value <0.09).

**Table 3.** Item Response Theory Parameter Estimates for the 11 Substance Use Disorder Criteria Assessed on the Substance Use Symptom Checklist Among Primary Care Patients Who Reported Daily Cannabis Use Only, Other Drug Use Only, and Both Daily Cannabis and Other Drug Use on Routine Screening From March 1, 2015, to March 1, 2020^a^

Substance Use Symptom Checklist item	Daily cannabis use only (n = 16 140)			Other drug use only (n = 4791)			Daily cannabis & other drug use (n = 2373)		
	Loading, λ	Discrimination, *a *(95% CI)	Severity, *b *(95% CI)	Loading, λ	Discrimination, *a *(95% CI)	Severity, *b *(95% CI)	Loading, λ	Discrimination, *a *(95% CI)	Severity, *b *(95% CI)
Tolerance	0.64	1.42 (1.35-1.50)	1.21 (1.17-1.30)	0.85	2.79 (2.57-3.00)	1.00 (0.93-1.10)	0.67	1.55 (1.39-1.70)	0.31 (0.23-0.40)
Withdrawal	0.78	2.09 (1.96-2.20)	1.86 (1.79-1.90)	0.94	4.72 (4.29-5.20)	1.06 (0.97-1.20)	0.83	2.57 (2.30-2.90)	0.96 (0.87-1.10)
Larger/longer	0.83	2.57 (2.42-2.70)	1.60 (1.53-1.70)	0.93	4.46 (4.07-4.90)	0.88 (0.80-1.00)	0.87	3.02 (2.69-3.40)	0.72 (0.64-0.80)
Quit/control	0.81	2.37 (2.24-2.50)	1.57 (1.51-1.60)	0.92	4.02 (3.68-4.40)	1.02 (0.94-1.10)	0.85	2.70 (2.41-3.00)	0.83 (0.75-0.90)
Time spent	0.83	2.57 (2.40-2.80)	2.03 (1.93-2.10)	0.96	5.72 (5.13-6.40)	1.13 (1.02-1.30)	0.88	3.07 (2.73-3.50)	0.94 (0.84-1.00)
Physical or psychological problems	0.73	1.81 (1.71-1.90)	1.19 (1.15-1.20)	0.90	3.46 (3.18-3.80)	0.74 (0.68-0.80)	0.82	2.41 (2.16-2.70)	0.37 (0.30-0.40)
Neglect roles	0.86	2.84 (2.64-3.10)	2.02 (1.91-2.10)	0.94	4.67 (4.24-5.10)	1.09 (1.00-1.20)	0.91	3.61 (3.19-4.10)	0.97 (0.86-1.10)
Hazardous use	0.68	1.56 (1.46-1.70)	2.40 (2.31-2.50)	0.87	3.01 (2.75-3.30)	1.37 (1.27-1.50)	0.72	1.75 (1.56-2.00)	1.13 (1.03-1.20)
Social or interpersonal problems	0.77	2.05 (1.93-2.20)	1.71 (1.64-1.80)	0.93	4.41 (4.02-4.80)	0.86 (0.79-0.90)	0.85	2.78 (2.49-3.10)	0.69 (0.61-0.80)
Craving	0.78	2.09 (1.98-2.20)	1.26 (1.21-1.30)	0.94	4.47 (4.08-4.90)	0.87 (0.80-1.00)	0.81	2.32 (2.08-2.60)	0.40 (0.34-0.50)
Activities given up	0.84	2.64 (2.48-2.80)	1.82 (1.74-1.90)	0.95	5.13 (4.64-5.70)	1.08 (0.99-1.20)	0.88	3.09 (2.74-3.50)	0.92 (0.82-1.00)
Model fit indices									
CFI^b^	0.989			0.998			0.995		
RMSEA^c^	0.034			0.031			0.035		
SRMSR^c^	0.034			0.021			0.026		

Abbreviations: CFI, comparative fit index; RMSEA, root mean standard error of approximation; SRMSR, standardized root mean square residual.

^a^ Standardized factor loadings (λ) characterize the strength of the association between an item and latent substance use disorder; values can range from 0 to 1 with loadings of 0.40 or greater considered substantive. Discrimination (*a*) characterizes how well an item differentiates patients with higher vs lower substance use disorder severity; values can range from −∞ to ∞, but typically range from 0 to 2, with higher values indicative of better discrimination. Severity (*b*) characterizes the value along the continuum of latent substance use disorder severity at which an item best discriminates, which is also the level of latent substance use disorder severity needed for patients to have a 50% chance of endorsing the item; values can range from −∞ to ∞ but typically range from −3 to 3, with higher values endorsed at higher levels of substance use disorder severity.

^b^ A value greater than 0.95 indicates acceptable model fit.

^c^ A value less than 0.05 indicates acceptable model fit.

### Item Characteristics

**Figure 2.**
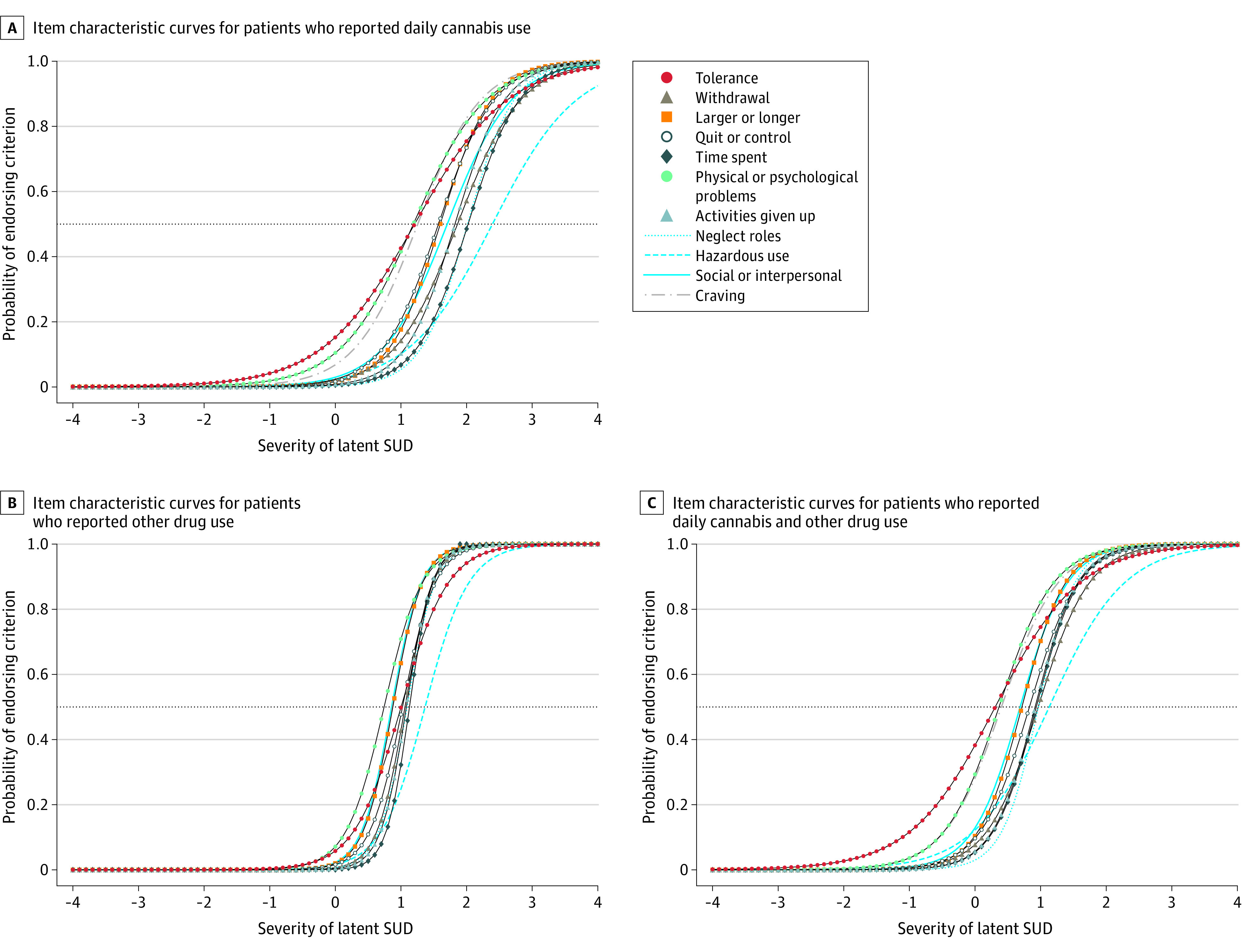
Item Characteristic Curves for 11 Substance Use Disorder (SUD) Criteria on the Substance Use Symptom Checklist Item characteristic curves among primary care patients who reported daily cannabis use only (n = 16 140) (A), other drug use only (n = 4791) (B), and both daily cannabis and other drug use (n = 2373) (C) on routine screening from March 1, 2015, to March 1, 2020. Each of the 11 criteria is represented as a separate curve. The slope of the curve corresponds to the discrimination parameter *a*. The point where each line intersects the dashed horizontal line indicates the level of latent SUD severity where patients have a 50% probability of endorsing the criterion, which corresponds to the severity parameter *b*.

For all symptom checklist subsamples, symptom checklist items discriminated higher vs lower SUD severity as expected (discrimination parameters all positive and significant) with the probability of endorsing each item increasing as SUD severity increased. While discrimination was higher than typical^33^ (illustrated by steep slopes in Figure 2), 1 item (tolerance) consistently did not discriminate as strongly as other items. Across subsamples, some items had lower severity parameters (eg, tolerance, physical or psychological problems, craving) and discriminated best when SUD severity was mild (illustrated by curves shifted further to the left in Figure 2). Other items had higher severity parameters (eg, hazardous use, time spent, neglect roles) and discriminated best when SUD was severe (illustrated by curves shifted further to the right in Figure 2).

### DIF and Clinical Impact

Several items had significant DIF by age and sex for all 3 symptom checklist subsamples. Additionally, there was significant DIF by race for patients who reported daily cannabis use only and by ethnicity for patients who reported other drug use only (eAppendices 3-5 in Supplement 1). However, correcting for item-level DIF changed expected SUD criteria counts by less than 0.5 criteria for patients with daily cannabis use only and less than 1 criterion for patients with other drug use or both daily cannabis and other drug use (eAppendix 6 in Supplement 1), indicating it was unlikely to significantly alter criteria counts that indicate SUD severity. Differences in comparative fit indices from models with and without correction for DIF suggested minimal impact (change in CFI <0.01) (eTable 10 in eAppendix 6 in Supplement 1).

## Discussion

This study evaluated the psychometric performance of a Substance Use Symptom Checklist used routinely among primary care patients reporting daily cannabis use, other drug use, or both. Consistent with *DSM-5* conceptualization of SUD, the symptom checklist measured SUD along a unidimensional continuum with items that discriminated SUD severity in each subsample. DIF analyses indicated that patient age, sex, race, and ethnicity did not meaningfully affect total criteria counts, suggesting minimal impact of DIF on accurately assessing SUD severity. Findings support use of the symptom checklist as a tool to aid clinicians in eliciting patient symptoms, identifying a spectrum of SUD severity, and supporting clinical decision-making based on *DSM-5* criteria.

To our knowledge, this is the first study to evaluate a Substance Use symptom checklist of *DSM-5* criteria when used routinely in primary care; however, findings are consistent with psychometric studies of SUD criteria in epidemiology studies^31,46,47,48,49,50,51,52,53,54,55,56,57^ and clinical trials.^31,58,59,60,61,62,63,64,65,66,67,68,69,70^ Psychometric studies were instrumental in the decision to revise *DSM* criteria, from abuse and dependence (*DSM-IV*) to a single SUD represented by a continuum of severity from 2 to 11 symptoms (*DSM-5*).^4,31^ Our findings build on this work to support use of the symptom checklist as a tool for assessing the spectrum of *DSM-5* SUD in primary care. While beyond the scope of this study, exploratory psychometric studies^71^ could improve on *DSM-5* conceptualization of SUD.

SUD symptoms represent negative consequences of use, which can be used in clinical conversations to help patients identify reasons for reducing substance use. Consistent with prior studies, tolerance was less discriminating than other criteria,^31^ particularly for patients who use cannabis only. This may reflect physiological adaptation to regular but not necessarily harmful use, and in the absence of 2 additional criteria, experts consider tolerance less likely to indicate SUD.^31^ Consistent with a previous study evaluating the psychometric performance of an Alcohol Symptom Checklist used in routine care,^44^ more discriminating items included neglect roles, time spent, and activities given up, which may reflect life domains that are important to patients.

Measurement is essential for improving care for patients using cannabis and other drugs.^25^ Implementation of symptom checklists for patients who self-report daily cannabis and/or other drug use, is affordable,^27^ feasible,^23,24,26^ and acceptable to patients in primary care.^5,25^ The ability to measure SUD symptoms can help with SUD identification, symptom management, and treatment planning for patients.^72^ Study findings support the symptom checklist’s construct validity^73^ as a scaled measure of SUD severity. The strong psychometric performance identified may help clinicians feel confident in measurement-based tools to support SUD identification and care in general medical settings where they are underrecognized and undertreated.^6,8,9^ A lack of clinically meaningful difference between expected total scores on the symptom checklist for patients of different ages, sexes, races, and ethnicities supports its use in diverse patient populations. Future studies are needed to evaluate test-retest reliability of the symptom checklist and discriminative validity compared with a confidential interview comparison standard for SUD.

### Limitations

The use of routinely collected assessment data documented in EHRs is a unique strength of this study; it also introduced important limitations. Symptom checklists were only routinely administered to those reporting “daily or almost daily” cannabis or any other drug use. Nevertheless, we identified a spectrum of SUDs in this patient population. For patients who reported daily cannabis and other drug use, it was unclear which substance predominantly contributed to symptoms. Similarly, among patients who reported other drug use, it was unknown what class of drugs (eg, opioids, stimulants) contributed to symptoms. However, for the symptom checklist to be practical in primary care, it was not possible to use different checklists for specific substances. Some patients may have underestimated or underreported substance use on behavioral health screens and/or symptoms on symptom checklists, although many patients were willing to report substance use and multiple SUD symptoms. Dichotomous endorsement of symptoms may not reflect the reality of how patients experience an SUD, and the symptom checklist did not assess the frequency of criteria; clinicians must use clinical judgment and confirm symptoms are recurrent to diagnose SUD. Nevertheless, the symptom checklist can prompt a patient-clinician conversation leading to diagnosis of specific SUDs. Additionally, this study was conducted in a predominantly non-Hispanic, White patient population insured by an integrated health care system in a US state where adult cannabis use is legal. Analyses of DIF by race may have been underpowered,^74^ and findings may not generalize to other patient populations, health systems, and settings. Despite these limitations, the use of data collected as part of routine care is an important and novel strength that lends external validity to findings.

## Conclusions

This cross-sectional study supports the Substance Use Symptom Checklist as a measure of SUD severity when used routinely in primary care. Specifically, the 11-item symptom checklist provided scaled, unidimensional information to help clinicians assess SUD symptoms and severity among patients with high-risk cannabis and other drug use. The symptom checklist was brief, feasible to administer in primary care, and performed equitably across age, sex, race, and ethnicity. These findings support the clinical utility of the symptom checklist as a tool for primary care.

## Notes

Supplement 1. **eFigure 1.** Routine Behavioral Health Screen With Single Items for Cannabis and Other Drugs **eFigure 2.** The Substance Use Symptom Checklist (Symptom Checklist): A *DSM-5* SUD Symptom Assessment Tool **eAppendix 1.** Detailed Description of Item Characteristics **eAppendix 2.** Detailed Description of Differential Item Functioning (DIF) Analyses **eAppendix 3.** DIF Findings for Patients Who Reported Daily Cannabis Use Only **eAppendix 4.** DIF Findings for Patients Who Reported Other Drug Use Only **eAppendix 5.** DIF Findings for Patients Who Reported Both Daily Cannabis and Other Drug Use **eAppendix 6.** Detailed Description of the Clinical Impact DIF Has on Estimated SUD Severity **eAppendix 7.** R Code for IRT and DIF Analyses
eReferences.

Supplement 2.
Data Sharing Statement
