# Supplementary material for: Psychometric Performance of a Substance Use Symptom Checklist to Help Clinicians Assess Substance Use Disorder in Primary Care
Source: JAMA Netw Open. 2023 May 26;6(5):e2316283. doi: 10.1001/jamanetworkopen.2023.16283 (PMC10220521; doi:10.1001/jamanetworkopen.2023.16283)
Supplement: Supplement 1. — eFigure 1. Routine Behavioral Health Screen With Single Items for Cannabis and Other Drugs eFigure 2. The Substance Use Symptom Checklist (Symptom Checklist): A DSM-5 SUD Symptom Assessment Tool eAppendix 1. Detailed Description of Item Characteristics eAppendix 2. Detailed Description of Differential Item Functioning (DIF) Analyses eAppendix 3. DIF Findings for Patients Who Reported Daily Cannabis Use Only eAppendix 4. DIF Findings for Patients Who Reported Other Drug Use Only eAppendix 5. DIF Findings for Patients Who Reported Both Daily Cannabis and Other Drug Use eAppendix 6. Detailed Description of the Clinical Impact DIF Has on Estimated SUD Severity eAppendix 7. R Code for IRT and DIF Analyses eReferences. [file jamanetwopen-e2316283-s001.pdf]

Supplemental Online Content

Matson TE, Hallgren KA, Lapham GT, et al. Psychometric performance of a substance use symptom checklist to help clinicians assess substance use disorder in primary care. *JAMA Netw Open.* 2023;6(5):e2316283. doi:10.1001/jamanetworkopen.2023.16283

eFigure 1. Routine Behavioral Health Screen With Single Items for Cannabis and Other Drugs

eFigure 2. The Substance Use Symptom Checklist (Symptom Checklist): A *DSM-5* SUD Symptom Assessment Tool

eAppendix 1. Detailed Description of Item Characteristics

eAppendix 2. Detailed Description of Differential Item Functioning (DIF) Analyses

eAppendix 3. DIF Findings for Patients Who Reported Daily Cannabis Use Only

eAppendix 4. DIF Findings for Patients Who Reported Other Drug Use Only

eAppendix 5. DIF Findings for Patients Who Reported Both Daily Cannabis and Other Drug Use

eAppendix 6. Detailed Description of the Clinical Impact DIF Has on Estimated SUD Severity

eAppendix 7. R Code for IRT and DIF Analyses

eReferences.

This supplemental material has been provided by the authors to give readers additional information about their work.

© 2023 Matson TE et al. *JAMA Network Open.*

eFigure 1. Routine Behavioral Health Screen With Single Items for Cannabis and Other Drugs

Over the past 2 weeks, how often have you been bothered by any of the following problems:

1. Little interest or pleasure in doing things?	**Not at all** 0	**Several days** 1	**More than half the days** 2	**Nearly every day** 3
2. Feeling down, depressed, or hopeless?	**Not at all** 0	**Several days** 1	**More than half the days** 2	**Nearly every day** 3

In the past year…

3. How often did you have a drink containing alcohol in the past year?	**Never** 0	**Monthly or less** 1	**2 to 4 times a month** 2	**2 to 3 times a week** 3	**4 or more times a week** 4

4. How many drinks containing alcohol did you have on a typical day when you were drinking in the past year?	**None** 0	**1 or 2 drinks** 0	**3 or 4 drinks** 1	**5 or 6 drinks** 2	**7 to 9 drinks** 3	**10 or more drinks** 4

5. How often did you have 6 or more drinks on one occasion in the past year?	**Never** 0	**Less than monthly** 1	**Monthly** 2	**Weekly** 3	**Daily or almost daily** 4
6. How often in the past year have you used marijuana?	**Never** 0	**Less than monthly** 1	**Monthly** 2	**Weekly** 3	**Daily or almost daily** 4
7. How often in the past year have you used an illegal drug (not marijuana) or used a prescription medication for non-medical reasons?	**Never** 0	**Less than monthly** 1	**Monthly** 2	**Weekly** 3	**Daily or almost daily** 4

Caption: The annual Behavioral Health Questionnaire includes single items for cannabis (#6) and any other drug use (#7). The questionnaire is prefaced with "*Once a year, we ask all our patients to complete this form on conditions that affect their health. Please help us provide you with the best medical care by answering the questions below*.

© 2023 Matson TE et al. *JAMA Network Open.*

eFigure 2. The Substance Use Symptom Checklist (Symptom Checklist): A *DSM-5* SUD Symptom Assessment Tool

Substance Use Symptom Checklist

KAISER PERMANENTE®

This checklist will help you and your provider understand how using marijuana or other drugs might be affecting your health.

Please think about your life in the 12 months. Then go through the questions below and answer "yes" or "no" for each one.

Patient Label

Name: ______________________

MRN: ______________________

Birth Date (MM/DD/YY): ____________

In the last 12 months…

1. Did using the same amount of the drug have less effect than it used to? Or did you have to use more to feel the effect you wanted? *Please answer "yes" if either question is true for you.*	No	Yes
2. Did you have withdrawal symptoms when you weren't using the drug? Or did you use the drug to avoid having these symptoms? *Please answer "yes" if either question is true for you.*	No	Yes
3. Did you have times when you used the drug more or for longer than you wanted to?	No	Yes
4. Did you want to cut back or stop using the drug, but couldn't?	No	Yes
5. Did you spend a lot of time trying to get the drug, using the drug, or recovering from using it?	No	Yes
6. Did you continue to use the drug even though you thought it might be causing mental or physical problems–or making them worse?	No	Yes
7. Did using the drug make it harder for you to keep up with your responsibilities at work, school, or home?	No	Yes
8. Did you do something dangerous more than once after using the drug–like drive a car or operate machinery?	No	Yes
9. Did you use the drug even though you thought it might be causing problems with your family or other people?	No	Yes
10. Did you have strong desires or cravings for the drug?	No	Yes
11. Did you spend less time working, enjoying hobbies, or being with others because of your use of the drug?	No	Yes

Which drug(s) did you use in the last year? Please circle all that apply.

- Opiates, including heroin
- Marijuana or cannabis
- Meth or other stimulants
- Cocaine
- Benzodiazepines or other sedatives
- Other: ______________________

BH4143000-01-20 © 2017 Kaiser Foundation Health Plan of Washington

© 2023 Matson TE et al. *JAMA Network Open.*

Caption: The Substance Use Symptom Checklist is prefaced with: "*To help you and your provider understand how your marijuana or other drug use might be affecting your health, please complete the following questions.*" Even though patients could circle substances that contribute to symptom burden, this information was missing for more than 50% of Symptom Checklists, which is why analyses stratified findings based on responses to cannabis and other drug screens that prompted assessment with the Symptom Checklist.

Abbreviations: DSM-5: Diagnostic and Statistical Manual of Mental Disorders, 5th Edition

© 2023 Matson TE et al. *JAMA Network Open.*

eAppendix 1. Detailed Description of Item Characteristics

Daily cannabis only

All items had high discrimination parameters,[1] ranging from 1.42 (tolerance) to 2.84 (neglect roles), demonstrating a strong association with SUD severity. Severity parameter ranged from 1.19 (physical/psychological problems) to 2.40 (hazardous use). Items with lower severity parameters (e.g., tolerance, physical/psychological problems, craving) discriminated best when latent SUD was mild whereas items with higher severity parameters (e.g., time spent, neglect roles, and hazardous use) discriminated best when latent SUD was severe. See Table 3 for item parameters and Figure 2 for item characteristic curves among patients who reported daily cannabis use only.

Other drug use only

All items had extremely high discrimination parameters,[1] ranging from 2.79 (tolerance) to 5.72 (time spent) and severity parameters ranging from 0.74 (physical/psychological problems) to 1.37 (hazardous use). One item (physical/psychological problems) discriminated best when latent SUD was mild, and two items discriminated best when latent SUD was severe (time spent, hazardous use) See Table 3 for item parameters and Figure 2 for item characteristic curves among patients who reported other drug use only.

Daily cannabis and other drug use

All items had high discrimination parameters,[1] ranging from 1.55 (tolerance) to 3.61 (neglect roles) and severity parameters ranging from 0.31 (tolerance) to 1.13 (hazardous use). As with the cannabis-only subsample, some items (tolerance, physical/psychological problems, craving) discriminated best when latent SUD was mild whereas other items discriminated best when latent SUD was severe (withdrawal, time spent, neglect roles, hazardous use, and activities given up). See Table 3 for item parameters and Figure 2 item characteristic curves among patients who reported both daily cannabis and other drug use.

© 2023 Matson TE et al. *JAMA Network Open.*

eAppendix 2. Detailed Description of Differential Item Functioning (DIF) Analyses

For each subsample (patients who reported daily cannabis only, other drug use only, both daily cannabis and other drug use), we tested for differential item functioning (DIF) by demographic factors and then examined the impact DIF had on the clinical utility of the Substance Use Symptom Checklist, as previously done for psychometric evaluation of an Alcohol Symptom Checklist.[2] Specifically, we tested whether item-level severity and discrimination parameters differed by age, sex, race, and ethnicity using a likelihood ratio test that compared a more complex model where item parameters were estimated separately (i.e., freely estimated) for each demographic subgroup to a simpler model that assumes item parameters are the same for subgroups.[3, 4] In the freely estimated model, discrimination and severity parameters were freely estimated for all items, with the exception of three "anchor" items for which we constrained parameters to be equal across demographic subgroups so that differences in the latent means and variances could be estimated between groups (e.g., male and female patients) without biasing DIF tests.[4, 5] We selected the three most consistently discriminating items[4] across the three subsamples to be anchor items: time spent, neglect roles, and activities given up. The most populous subgroup in each demographic category was selected as the reference group with latent means set at 0 and latent variances set at 1. Latent means and variances were freely estimated in other subgroups. We used an alpha level of 0.05/11 items to account for multiple comparisons. DIF results for each demographic subgroup within each subsample are presented in Supplemental Tables 1-8.

DIF may be present without having clinically meaningful impact on the performance of the Substance Use Symptom Checklist. For example, DIF may be present in opposite directions for different items, effectively canceling out.[3] Additionally, DIF may be present in small amounts but still statistically significant due to a large sample size.[6] Because the total number of symptoms (i.e., DSM-5 criteria) endorsed on the Substance Use Symptom Checklist is used by clinicians to determine the presence and severity of SUD, it is useful to examine the impact of DIF on the total expected number (0-11) of criteria endorsed. Within each subsample, we used the IRT model that freely estimated item parameters to calculate: 1) the maximum difference between subgroups at any point along the severity continuum, and 2) the maximum difference between subgroups at mild (2-3 symptoms), moderate (4-5 symptoms), and severe (≥6 symptoms) thresholds of SUD,[7] which could affect clinical decision-making regarding diagnosis and treatment of SUD. Differences are summarized in the main paper, presented in Table 9, and graphically illustrated in Figures 3-5.

Lastly, we compared freely estimated models with correction for DIF and constrained models without correction for DIF to determine whether any item-level DIF led to meaningful differences. A difference in comparative fit indices (CFI) value >0.01 has been proposed[8] as another method for determining if there is meaningful DIF on the absolute model fit of the factor analysis. Differences in fit indices are presented in Table 10.

eAppendix 3. DIF Findings for Patients Who Reported Daily Cannabis Use Only

Among the subsample who reported daily cannabis uses only, there was significant DIF associated with age (6 items), sex (3 items), and race (2 items), but not Hispanic ethnicity.

By age

Six items (tolerance, withdrawal, physical/psychological problems, hazardous use, social/interpersonal problems, and craving) had significant differential item functioning by age for both the discrimination and severity parameters.

In addition to DIF, there were differences in latent means and variances between some age groups. Patients age 18-24 had, on average, higher SUD severity (latent mean >0) than patients 25-44, whereas patients 45-64 and 65+ had, on average, lower SUD severity (latent mean <0) than patients 25-44. For all age groups, latent SUD was less variable (latent variance <1) than patients age 25-44.

eTable 1. Differential item functioning (DIF) by age for primary care patients who reported daily cannabis use only on routine screening March 2015-March 2020 (n=16,140)

		25-44*		18-24		45-64		65+	
DSM-5 Checklist Item		*a*	*b*	*a*	*b*	*a*	*b*	*a*	*b*
1)	Tolerance	1.29	1.2	1.33	0.95	1.49	1.34	1.6	1.34
2)	Withdrawal	2.03	1.79	2.16	1.97	2.13	1.71	2.42	1.87
3)	Larger/longer	2.53	1.57	–	–	–	–	–	–
4)	Quit/control	2.32	1.55	–	–	–	–	–	–
5)	Time spent†	2.57	1.99	–	–	–	–	–	–
6)	Physical/psychological problems	2.02	1.21	1.74	1.28	1.93	0.90	1.69	0.85
7)	Neglect roles†	2.81	1.99	–	–	–	–	–	–
8)	Hazardous use	1.52	2.5	1.47	2.39	1.76	2.09	1.6	2.06
9)	Social/interpersonal problems	2.20	1.77	1.75	1.66	2.19	1.49	2.20	1.40
10)	Craving	1.95	1.21	1.94	1.18	2.18	1.30	2.80	1.25
11)	Activities given up†	2.56	1.81	–	–	–	–	–	–
Full Test Parameters									
	Latent mean	0.00		0.38		-0.27		-0.43	
	Latent variance	1.00		0.93		0.92		0.90	

Note. * = reference group, † = anchor item, *a* = discrimination parameter estimate, *b* = severity parameter estimate. Item parameters that significantly differed from the reference group are presented in the table; item parameters that did not significantly differ from the reference group or that were fixed as anchoring items are indicated with dashes (–). Latent means and variances were fixed to 0 and 1, respectively, for the reference group and were freely estimated for non-reference groups.

© 2023 Matson TE et al. *JAMA Network Open.*

By sex

Three items (larger/longer, hazardous use, craving) had significant differential item functioning by sex for both discrimination and severity parameters.

In addition to DIF, female patients had lower and more variable SUD severity (latent mean <0 and latent variance >1), on average, than male patients.

eTable 2. Differential item functioning (DIF) by sex for primary care patients who reported daily cannabis use only on routine screening March 2015-March 2020 (n=16,140)

		Male*		Female	
DSM-5 Checklist Item		*a*	*b*	*a*	*b*
1)	Tolerance	1.39	1.11	–	–
2)	Withdrawal	2.04	1.77	–	–
3)	Larger/longer	2.48	1.57	2.65	1.40
4)	Quit/control	2.31	1.48	–	–
5)	Time spent†	2.51	1.94	–	–
6)	Physical/psychological problems	1.77	1.08	–	–
7)	Neglect roles†	2.78	1.93	–	–
8)	Hazardous use	1.44	2.24	1.67	2.45
9)	Social/interpersonal problems	2.00	1.61	–	–
10)	Craving	2.04	1.21	2.08	1.07
11)	Activities given up†	2.60	1.73	–	–
Full Test Parameters					
	Latent mean	0.00		0.28	
	Latent variance	1.00		1.07	

Note. * = reference group, † = anchor item, *a* = discrimination parameter estimate, *b* = severity parameter estimate. Item parameters that significantly differed from the reference group are presented in the table; item parameters that did not significantly differ from the reference group or that were fixed as anchoring items are indicated with dashes (–). Latent means and variances were fixed to 0 and 1, respectively, for the reference group and were freely estimated for non-reference groups.

© 2023 Matson TE et al. *JAMA Network Open.*

By race

One item (quit/control) had significant DIF by race for the discrimination parameter and two items (tolerance, quit/control) had significant DIF for the severity parameter. Analyses may have been underpowered to detect DIF given small numbers for some races (see Table 1).

In addition to DIF, American Indian/Alaska Native, Asian, Black/African American, and Native Hawaiian/Pacific Islander patients had, on average, higher and less variable SUD severity (latent mean >0; latent variance <1) than White patients.

eTable 3. Differential item functioning (DIF) by race for primary care patients who reported daily cannabis use only on routine screening March 2015-March 2020 (n=16,140)

		White *		American Indian / Alaska Native		Asian/Asian American		Black / African American		Hawaiian / Pacific Islander	
DSM-5 Checklist Item		*a*	*b*	*a*	*b*	*a*	*b*	*a*	*b*	*a*	*b*
1)	Tolerance	1.45	1.22	–	1.49	–	1.00	–	1.43	–	1.27
2)	Withdrawal	2.13	1.89	–	–	–	–	–	–	–	–
3)	Larger/longer	2.58	1.65	–	–	–	–	–	–	–	–
4)	Quit/control	2.44	1.63	2.86	1.36	2.00	1.67	2.33	1.38	3.90	1.42
5)	Time spent†	2.59	2.05	–	–	–	–	–	–	–	–
6)	Physical/psychological problems	1.83	1.20	–	–	–	–	–	–	–	–
7)	Neglect roles†	3.00	2.02	–	–	–	–	–	–	–	–
8)	Hazardous use	1.60	2.41	–	–	–	–	–	–	–	–
9)	Social/interpersonal problems	2.11	1.72	–	–	–	–	–	–	–	–
10)	Craving	2.12	1.29	–	–	–	–	–	–	–	–
11)	Activities given up†	2.69	1.84	–	–	–	–	–	–	–	–
Full Test Parameters											
	Latent mean	0.00		0.17		0.33		0.26		0.11	
	Latent variance	1.00		0.77		0.95		0.78		0.79	

Note. * = reference group, † = anchor item, *a* = discrimination parameter estimate, *b* = severity parameter estimate. Item parameters that significantly differed from the reference group are presented in the table; item parameters that did not significantly differ from the reference group or that were fixed as anchoring items are indicated with dashes (–). Latent means and variances were fixed to 0 and 1, respectively, for the reference group and were freely estimated for non-reference groups.

By ethnicity

There was no DIF by ethnicity although analyses may have been underpowered due to small numbers of some subgroups to detect any differences.

© 2023 Matson TE et al. *JAMA Network Open.*

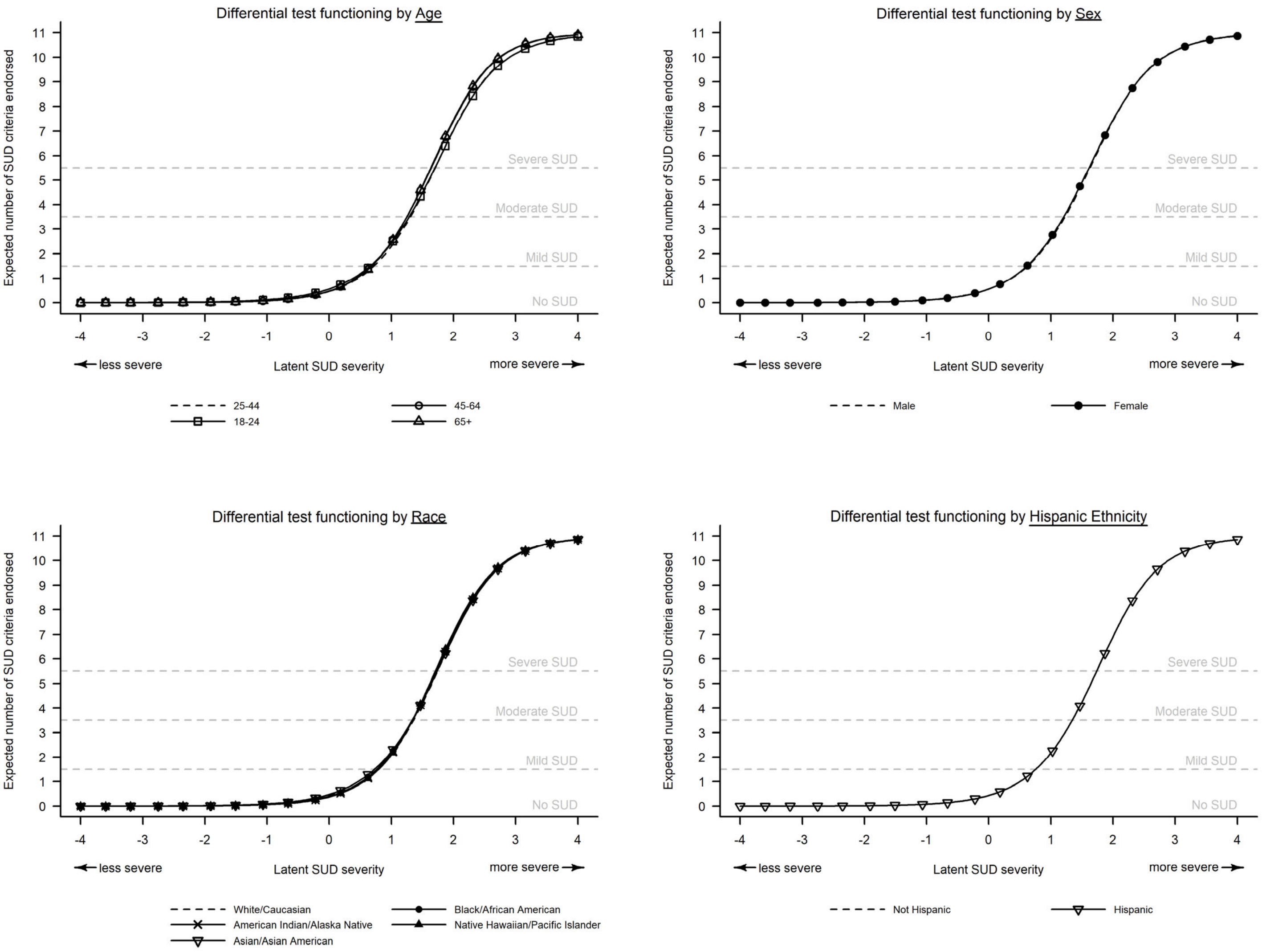

eFigure 3**.** Cumulative impact of differential item functioning (DIF) across age, sex, race, and ethnicity for primary care patients who reported daily cannabis use only on routine screening March 2015-March 2020 (n=16,140)

Caption: Using freely estimated models that corrected for DIF, the total expected number of SUD criteria endorsed on the Substance Use Symptom Checklist (y-axis) was plotted as a function of latent SUD severity (x-axis) for each subgroup. The vertical distances between curves represent the difference in total expected scores between subgroups with the same latent SUD severity. This difference was small, as indicated by test characteristic curves that nearly overlap, and never diverged more than half of one criterion indicating that DIF had minimal cumulative impact.

© 2023 Matson TE et al. *JAMA Network Open.*

eAppendix 4. DIF Findings for Patients Who Reported Other Drug Use Only

Among the subsample who reported other drug use only, there was significant DIF associated with age (2 items), sex (1 item), and ethnicity (1 items), but not race.

By age

Two items (tolerance and quit/control) had significant DIF by age for both discrimination and severity parameters.

In addition to DIF, patients 18-24 and 45-64 had higher SUD severity (latent mean >0) but severity was less variable (latent variance <1) than patients 25-44. Patients 65 and over had lower (latent mean <0) and less variable (latent variance <1) SUD severity.

eTable 4. Differential item functioning (DIF) by age for primary care patients who reported other drug use only on routine screening March 2015-March 2020 (n=4,791)

		25-44*		18-24		45-64		65+	
DSM-5 Checklist Item		*a*	*b*	*a*	*b*	*a*	*b*	*a*	*b*
1)	Tolerance	3.1	1.03	3.21	0.85	3.12	1.01	5.37	0.79
2)	Withdrawal	5.25	1.02	–	–	–	–	–	–
3)	Larger/longer	4.97	0.85	–	–	–	–	–	–
4)	Quit/control	4.13	0.99	5.55	1.06	4.47	0.88	7.89	0.74
5)	Time spent†	6.36	1.08	–	–	–	–	–	–
6)	Physical/psychological problems	3.86	0.74	–	–	–	–	–	–
7)	Neglect roles†	5.19	1.05	–	–	–	–	–	–
8)	Hazardous use	3.35	1.3	–	–	–	–	–	–
9)	Social/interpersonal problems	4.92	0.84	–	–	–	–	–	–
10)	Craving	5.02	0.85	–	–	–	–	–	–
11)	Activities given up†	5.75	1.04	–	–	–	–	–	–
Full Test Parameters									
	Latent mean	0.00		0.24		0.05		-0.14	
	Latent variance	1.00		0.57		0.77		0.44	

Note. * = reference group, † = anchor item, *a* = discrimination parameter estimate, *b* = severity parameter estimate. Item parameters that significantly differed from the reference group are presented in the table; item parameters that did not significantly differ from the reference group or that were fixed as anchoring items are indicated with dashes (–). Latent means and variances were fixed to 0 and 1, respectively, for the reference group and were freely estimated for non-reference groups.

© 2023 Matson TE et al. *JAMA Network Open.*

By sex

One item (hazardous use) had significant DIF by sex for both discrimination and severity parameters.

In addition to DIF, female patients had slightly lower (latent mean <0) and more variable SUD severity (latent variance >1) than male patients.

eTable 5. Differential item functioning (DIF) by sex for primary care patients who reported other drug use only on routine screening March 2015-March 2020 (n=4,791)

	Male*		Female	
DSM-5 Checklist Item	*a*	*b*	*a*	*b*
1) Tolerance	2.64	0.98	–	–
2) Withdrawal	4.48	1.04	–	–
3) Larger/longer	4.22	0.85	–	–
4) Quit/control	3.81	1	–	–
5) Time spent[†]	5.42	1.11	–	–
6) Physical/psychological problems	3.28	0.71	–	–
7) Neglect roles[†]	4.42	1.08	–	–
8) Hazardous use	2.78	1.3	3.11	1.45
9) Social/interpersonal problems	4.18	0.83	–	–
10) Craving	4.26	0.84	–	–
11) Activities given up[†]	4.87	1.06	–	–
Full Test Parameters				
Latent mean	0.00		0.19	
Latent variance	1.00		1.29	

Note. * = reference group, † = anchor item, *a* = discrimination parameter estimate, *b* = severity parameter estimate. Item parameters that significantly differed from the reference group are presented in the table; item parameters that did not significantly differ from the reference group or that were fixed as anchoring items are indicated with dashes (–). Latent means and variances were fixed to 0 and 1, respectively, for the reference group and were freely estimated for non-reference groups.

© 2023 Matson TE et al. *JAMA Network Open.*

By race

There was no DIF by race although analyses may have been underpowered to detect any differences due to small numbers of some subgroups.

By ethnicity

One item (craving) had significant DIF by Hispanic ethnicity for the severity parameter.

In addition to DIF, Hispanic patients had, on average, higher (latent mean >0) and less variable (latent variance <1) SUD severity than not Hispanic patients.

eTable 6. Differential item functioning (DIF) by ethnicity for primary care patients who reported other drug use only on routine screening March 2015-March 2020 (n=4,791)

	Not Hispanic*		Hispanic	
DSM-5 Checklist Item	*a*	*b*	*a*	*b*
1) Tolerance	2.83	1.01	–	–
2) Withdrawal	4.76	1.05	–	–
3) Larger/longer	4.51	0.88	–	–
4) Quit/control	4.03	1.02	–	–
5) Time spent†	5.78	1.13	–	–
6) Physical/psychological problems	3.52	0.75	–	–
7) Neglect roles†	4.79	1.09	–	–
8) Hazardous use	3.07	1.37	–	–
9) Social/interpersonal problems	4.49	0.86	–	–
10) Craving	4.53	0.86	–	1.02
11) Activities given up†	5.21	1.08	–	–
Full Test Parameters				
Latent mean	0.00		0.11	
Latent variance	1.00		0.74	

Note. * = reference group, † = anchor item, *a* = discrimination parameter estimate, *b* = severity parameter estimate. Item parameters that significantly differed from the reference group are presented in the table; item parameters that did not significantly differ from the reference group or that were fixed as anchoring items are indicated with dashes (–). Latent means and variances were fixed to 0 and 1, respectively, for the reference group and were freely estimated for non-reference groups.

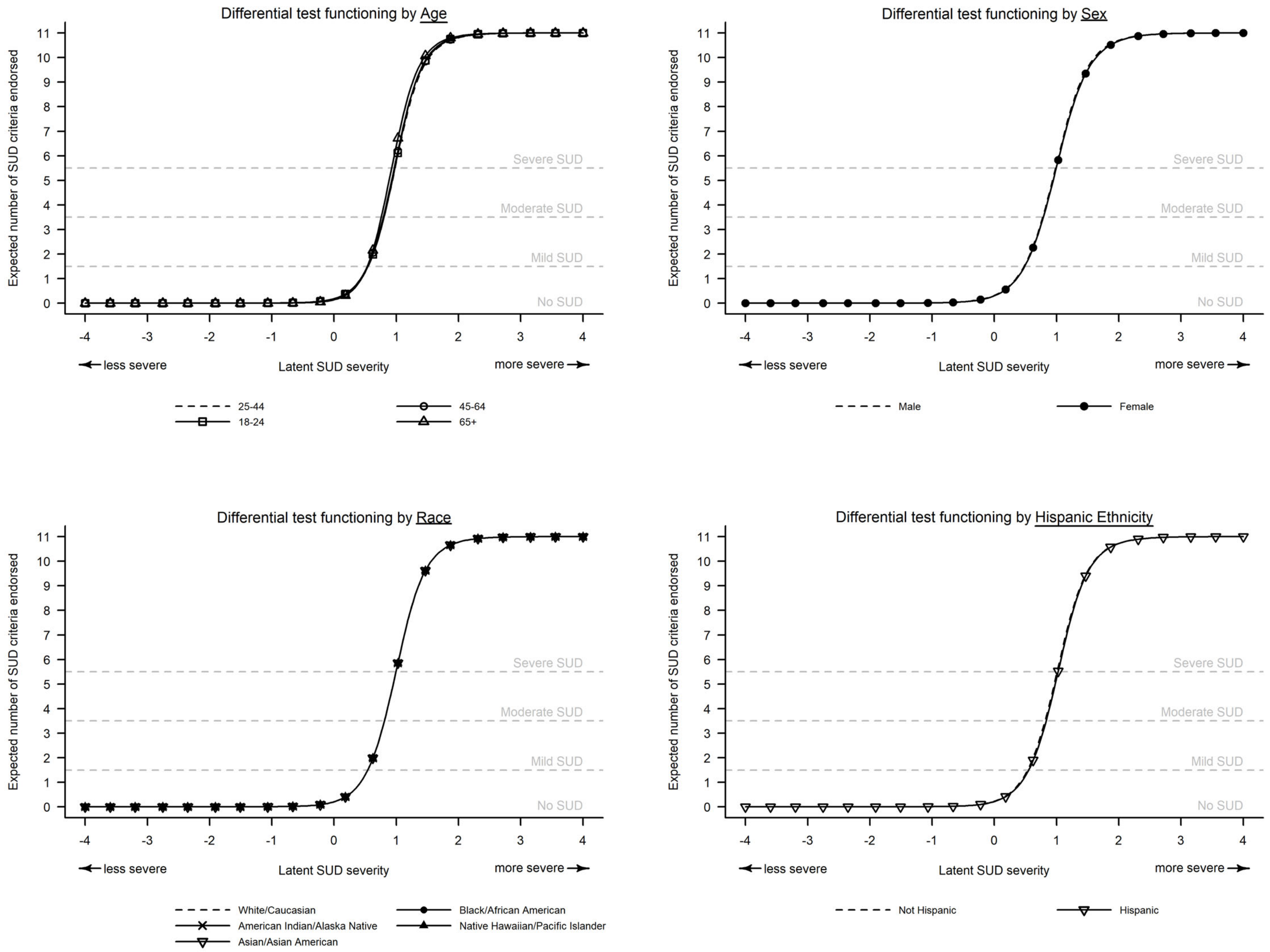

eFigure 4. Cumulative impact of differential item functioning (DIF) across age, sex, race, and ethnicity for primary care patients who reported other drug use only on routine screening March 2015-March 2020 (n=4,791)

Caption: Using freely estimated models that corrected for DIF, the total expected number of SUD criteria endorsed on the Substance Use Symptom Checklist (y-axis) was plotted as a function of latent SUD severity (x-axis) for each subgroup. The vertical distances between curves represent the difference in total expected scores between subgroups with the same latent SUD severity. This difference was small, as indicated by test characteristic curves that nearly overlap, and never diverged more than 2/3 of one criterion, indicating that DIF had minimal cumulative impact.

© 2023 Matson TE et al. *JAMA Network Open.*

eAppendix 5. DIF Findings for Patients Who Reported Both Daily Cannabis and Other Drug Use

Among the subsample of patients who reported both daily cannabis use and other drug use, there was significant DIF associated with age (1 item) and sex (1 item), but not race or ethnicity.

By age

One item (tolerance) had DIF by age for the discrimination parameter, and three items (tolerance, quit/control, craving) had DIF by age for the severity parameter.

In addition to DIF, patients 18-24 had, on average, higher SUD severity (latent mean >0) and less variable (latent variance <1) than patients 25-44 while patients 45-64 and 65+ had, on average, lower SUD severity (latent mean <0) than patients 25-44. Patients 45-64 had more variable SUD severity (latent mean>1) while patients 65+ had less variable SUD severity (latent mean<1) than patients 25-44.

eTable 7. Differential item functioning (DIF) by age for primary care patients who reported both daily cannabis use and other drug use on routine screening March 2015-March 2020 (n=2,373)

	25-44*		18-24		45-64		65+	
DSM-5 Checklist Item	*a*	*b*	*a*	*b*	*a*	*b*	*a*	*b*
1) Tolerance	1.46	0.5	1.74	0.21	1.61	0.96	1.92	0.69
2) Withdrawal	2.66	1.07	–	–	–	–	–	–
3) Larger/longer	3.09	0.83	–	–	–	–	–	–
4) Quit/control	2.87	0.9	–	1.04	–	0.7	–	0.96
5) Time spent†	3.14	1.05	–	–	–	–	–	–
6) Physical/psychological problems	2.48	0.49	–	–	–	–	–	–
7) Neglect roles†	3.72	1.07	–	–	–	–	–	–
8) Hazardous use	1.79	1.24	–	–	–	–	–	–
9) Social/interpersonal problems	2.85	0.81	–	–	–	–	–	–
10) Craving	2.37	0.49	–	0.5	–	0.65	–	1.3
11) Activities given up†	3.16	1.03	–	–	–	–	–	–
Full Test Parameters								
Latent mean	0.00		0.40		-0.02		-0.07	
Latent variance	1.00		0.76		1.23		0.34	

Note. * = reference group, † = anchor item, *a* = discrimination parameter estimate, *b* = severity parameter estimate. Item parameters that significantly differed from the reference group are presented in the table; item parameters that did not significantly differ from the reference group or that were fixed as anchoring items are indicated with dashes (–). Latent means and variances were fixed to 0 and 1, respectively, for the reference group and were freely estimated for non-reference groups.

© 2023 Matson TE et al. *JAMA Network Open.*

By age

One item (hazardous use) had significant DIF by sex for the severity parameter.

In addition to DIF, female patients had, on average, lower and more variable SUD severity (latent mean <0; latent variance >1) than male patients.

eTable 8. Differential item functioning (DIF) by sex for primary care patients who reported both daily cannabis use and other drug use on routine screening March 2015-March 2020 (n=2,373)

		Male*		Female	
DSM-5 Checklist Item		*a*	*b*	*a*	*b*
1)	Tolerance	1.46	0.28	–	–
2)	Withdrawal	2.44	0.98	–	–
3)	Larger/longer	2.85	0.71	–	–
4)	Quit/control	2.55	0.83	–	–
5)	Time spent†	2.87	0.95	–	–
6)	Physical/psychological problems	2.27	0.34	–	–
7)	Neglect roles†	3.42	0.98	–	–
8)	Hazardous use	1.68	1.01	–	1.42
9)	Social/interpersonal problems	2.62	0.68	–	–
10)	Craving	2.19	0.38	–	–
11)	Activities given up†	2.91	0.93	–	–
Full Test Parameters					
	Latent mean	0.00		0.13	
	Latent variance	1.00		1.35	

Note. * = reference group, † = anchor item, *a* = discrimination parameter estimate, *b* = severity parameter estimate. Item parameters that significantly differed from the reference group are presented in the table; item parameters that did not significantly differ from the reference group or that were fixed as anchoring items are indicated with dashes (–). Latent means and variances were fixed to 0 and 1, respectively, for the reference group and were freely estimated for non-reference groups.

By Race and Ethnicity

There was no significant DIF by race or ethnicity. Findings may have been underpowered to detect differences due to small numbers in some demographic subgroups.

© 2023 Matson TE et al. *JAMA Network Open.*

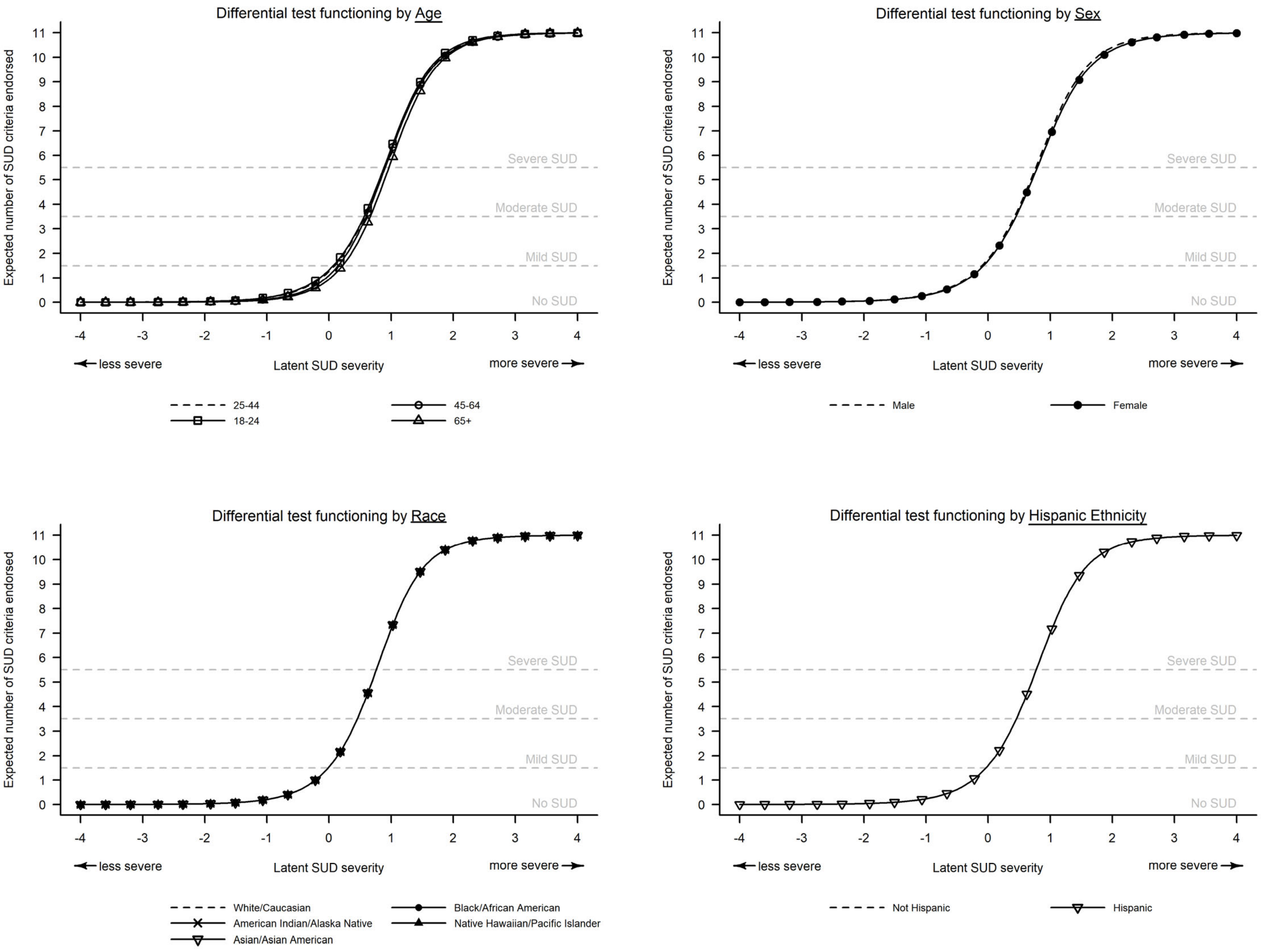

eFigure 5. Cumulative impact of differential item functioning (DIF) across age, sex, race, and ethnicity for primary care patients who reported both daily cannabis use and other drug use on routine screening March 2015-March 2020 (n=2,373)

Caption: Using freely estimated models that corrected for DIF, the total expected number of SUD criteria endorsed on the Substance Use Symptom Checklist (y-axis) was plotted as a function of latent SUD severity (x-axis) for each subgroup. The vertical distances between curves represents the difference in total expected scores between subgroups with the same latent SUD severity. This difference was small, as indicated by test characteristic curves that nearly overlap, and never diverged more than 2/3 of one criterion, indicating that DIF had minimal cumulative impact.

© 2023 Matson TE et al. *JAMA Network Open.*

eAppendix 6. Detailed Description of the Clinical Impact DIF Has on Estimated SUD Severity

Patients who reported daily cannabis use only

For patients who reported daily cannabis use only, the clinical impact of DIF was minimal in all cases. Differences in expected DSM-5 criteria counts for persons from different demographic subgroups with the same latent SUD severity were small and never diverged more than half of one criterion (Supplemental Figure 3), suggesting that differential item functioning had minimal cumulative impact on total criterion counts. When SUD severity was held constant, differential item functioning was expected to produce differences in SUD criteria that never exceeded 0.42 criteria (out of 11 possible) for age (patients 65+ reporting more criteria), 0.09 for sex (female patients reporting more criteria) 0.20 criteria for race (NH/PI patients reporting more criteria). These maximum differences tended to occur at high levels of latent SUD severity (e.g., more than 6 criteria). At clinical decision-making thresholds for mild, moderate, and severe SUD, differential item functioning was expected to produce even smaller differences (Supplemental Table 9). Further, comparing models with versus without correction for DIF did not improve model fit (ΔCFI<0.01; Supplemental Table 10). In other words, the gain in model fit by allowing parameters to be freely estimated for each demographic subgroup (versus constrained to be equal across demographic subgroups), was very small.

Patients who reported other drug use only

For patients who reported other drug use only, the clinical impact of DIF was also minimal. Differences in expected DSM-5 criteria counts for persons from different demographic subgroups with the same latent SUD severity never diverged by more than two thirds of one criterion (Supplemental Figure 4). When SUD severity was held constant, differential item functioning was expected to produce differences in SUD criteria that never exceeded 0.66 criteria (out of 11 possible) for age (patients 65+ reporting more criteria), 0.11 for sex (female patients reporting fewer criteria) 0.17 for ethnicity (Hispanic patients reporting fewer criteria). At clinical decision-making thresholds for mild, moderate, and severe SUD, the largest differences were expected at the severe threshold (Supplemental Table 9). Comparing models with versus without correction for DIF did not improve model fit (ΔCFI<0.01; Supplemental Table 10).

Patients who reported both daily Cannabis and other drug use

For patients who reported both daily cannabis and other drug use, the clinical impact of DIF was again minimal. Differences in expected DSM-5 criteria counts for persons from different demographic subgroups with the same latent SUD severity never diverged by more than two thirds of one criterion (Supplemental Figure 5). When SUD severity was held constant, differential item functioning was expected to produce differences in SUD criteria that never exceeded 0.57 criteria (out of 11 possible) for age (patients 65+ reporting fewer criteria) and 0.17 for sex (female patients reporting fewer criteria). At clinical decision-making thresholds for mild, moderate, and severe SUD, differential item functioning was expected to produce even smaller differences (Supplemental Table 9). As with the prior two subsamples, comparing models with versus without correction for DIF did not improve model fit (ΔCFI<0.01; Supplemental Table 10).

© 2023 Matson TE et al. *JAMA Network Open.*

eTable 9. Differences in expected DSM-5 criteria count at clinical decision-making thresholds for mild, moderate, and severe SUD among primary care patients who reported daily cannabis only, other drug use only, and both daily cannabis and other drug use on routine screening March 2015-March 2020

	**Daily Cannabis Only (n=16,140)**			**Other Drug Use Only (n=4,791)**			**Both Daily Cannabis and Other Drug Use (n=2,373)**		
	Mild	Moderate	Severe	Mild	Moderate	Severe	Mild	Moderate	Severe
Age									
18-24 vs. 25-44	0.13	0.09	0.03	0.01	0.01	0.04	0.06	0.04	0.02
18-24 vs. 45-64	0.03	-0.18	-0.32	-0.05	-0.11	-0.1	0.23	0.19	0.13
18-24 vs. 65+	0.05	-0.19	-0.36	-0.08	-0.48	-0.62	0.41	0.56	0.55
25-44 vs. 18-24	-0.13	-0.09	-0.03	-0.01	-0.01	-0.04	-0.06	-0.04	-0.02
25-44 vs. 45-64	-0.11	-0.27	-0.35	-0.06	-0.12	-0.15	0.17	0.14	0.11
25-44 vs. 65+	-0.09	-0.28	-0.39	-0.09	-0.49	-0.66	0.35	0.51	0.53
45-64 vs. 18-24	-0.03	0.18	0.32	0.05	0.11	0.1	-0.23	-0.19	-0.13
45-64 vs. 25-44	0.11	0.27	0.35	0.06	0.12	0.15	-0.17	-0.14	-0.11
45-64 vs. 65+	0.02	-0.01	-0.04	-0.03	-0.37	-0.52	0.18	0.37	0.42
65+ vs. 18-24	-0.05	0.19	0.36	0.08	0.48	0.62	-0.41	-0.56	-0.55
65+ vs. 25-44	0.09	0.28	0.39	0.09	0.49	0.66	-0.35	-0.51	-0.53
65+ vs. 45-64	-0.02	0.01	0.04	0.03	0.37	0.52	-0.18	-0.37	-0.42
Sex									
Female vs. Male	0.03	0.08	0.08	-0.04	-0.08	-0.1	-0.06	-0.12	-0.15
Male vs. Female	-0.03	-0.08	-0.08	0.04	0.08	0.1	0.06	0.12	0.15
Race									
AI/AN vs. Asian	-0.14	-0.02	0.06	–	–	–	–	–	–
AI/AN vs. Black	-0.05	-0.01	0.03	–	–	–	–	–	–
AI/AN vs. NH/PI	0.02	-0.02	0.1	–	–	–	–	–	–
AI/AN vs. White	-0.03	0.07	0.09	–	–	–	–	–	–
Asian vs. AI/AN	0.14	0.02	-0.06	–	–	–	–	–	–
Asian vs. Black	0.09	0.02	-0.03	–	–	–	–	–	–
Asian vs. NH/PI	0.16	0.01	-0.16	–	–	–	–	–	–
Asian vs. White	0.11	0.09	0.04	–	–	–	–	–	–
Black vs. AI/AN	0.05	0.01	-0.03	–	–	–	–	–	–
Black vs. Asian	-0.09	-0.02	0.03	–	–	–	–	–	–
Black vs. NH/PI	0.07	-0.01	-0.13	–	–	–	–	–	–
Black vs. White	0.02	0.07	0.06	–	–	–	–	–	–
NH/PI vs. AI/AN	-0.02	0.02	-0.1	–	–	–	–	–	–
NH/PI vs. Asian	-0.16	-0.01	0.16	–	–	–	–	–	–
NH/PI vs. Black	-0.07	0.01	0.13	–	–	–	–	–	–
NH/PI vs. White	-0.05	0.08	0.2	–	–	–	–	–	–
White vs. AI/AN	0.03	-0.07	-0.09	–	–	–	–	–	–
White vs. Asian	-0.11	-0.09	-0.04	–	–	–	–	–	–
White vs. Black	-0.02	-0.07	-0.06	–	–	–	–	–	–
White vs. NH/PI	0.05	-0.08	-0.2	–	–	–	–	–	–
Ethnicity									

© 2023 Matson TE et al. *JAMA Network Open.*

Hispanic vs. Not Hispanic	–	–	–	-0.08	-0.16	-0.17	–	–	–
Not Hispanic vs. Hispanic	–	–	–	0.08	0.16	0.17	–	–	–

Abbreviations: DSM-5=Diagnostic and Statistical Manual of Mental Disorders, 5th Edition; SUD=Substance Use Disorder; AI/AN=American Indian/Alaska Native; NH/PI=Native Hawaiian/Pacific Islander

Note. Subgroups that had no significant DIF have no expected differences in criteria count, indicated with dashes (–). A negative estimate suggests fewer expected criteria endorsed relative to the comparator group while a positive estimate suggests more expected criteria endorsed relative to the comparator group at the same level of latent SUD severity.

© 2023 Matson TE et al. *JAMA Network Open.*

eTable 10. Comparison of models with and without correction for differential item functioning (DIF)

	Constrained model[a] (No correction for DIF)	Freely-estimated model[b] (Correction for DIF)	
	CFI	CFI	ΔCFI[c]
Daily cannabis use only			
Age	0.978	0.986	0.008
Sex	0.987	0.988	0.002
Race	0.989	0.989	0.001
Ethnicity[d]	0.988	0.988	0.000
Other drug use only			
Age	0.997	0.998	0.001
Sex	0.998	0.998	0.000
Race[d]	0.998	0.998	0.000
Ethnicity	0.998	0.998	0.000
Both daily cannabis and other drug use			
Age	0.990	0.994	0.004
Sex	0.994	0.995	0.001
Race[d]	0.995	0.995	0.000
Ethnicity[d]	0.994	0.994	0.000

Abbreviation: CFI = comparative fit index; DIF=differential item functioning; Δ=difference

[a] IRT model parameters are constrained to be the same for demographic subgroups

[b] IRT model parameters are freely estimated for demographic subgroups

[c] If the difference in comparative fit indices (CFI) is <0.01, it suggests that DIF did not have a meaningful impact on absolute model fit.

[d] DIF was not detected for this subgroup

© 2023 Matson TE et al. *JAMA Network Open.*

eAppendix 7. R Code for IRT and DIF Analyses

```
# by Theresa Matson and Kevin Hallgren


## IRT ANALYSES ##

# Fit unidimensional IRT model
model <- mirt(data = dat[,paste0("aud___q",1:11)],
              model ="F1 = 1-11",
              itemtype = "2PL",
              SE = T,
              SE.type = "SEM",
              calcNull = T)

# Examine factor loadings
summary(model)

# Number and perecent endorsing each item
colSums(dat)
colMeans(dat)

# Discrimination and Severity parameters
coef(model, IRTpars = T, simplify = T, printSE = T)

# Confidence intervals
ci <- PLCI.mirt(model) # provides a, must convert to d to b

blb <- -((ci$upper_97.5[ci$parnam=="d"]) / (ci$value[ci$parnam=="a1"]))
bub <- -((ci$lower_2.5[ci$parnam=="d"]) / (ci$value[ci$parnam=="a1"]))
b_ci <- as.data.frame(t(rbind(blb, b, bub)))
b_ci

# Global fit indices
M2(model)

# Range of residuals
res.m = residuals(model)
range(res.m[upper.tri(res.m)])

# Eigenvalues
tetra = tetrachoric(dat)
eigen(tetra$rho)

# Basic ICC curve
plot(model, type = 'trace', facet_items = F, theta_lim=c(-4,4))
```

© 2023 Matson TE et al. *JAMA Network Open.*

```
## DIF Analyses ##

# Set specifications
groupvar = dat$sex
pcrit = 0.05/11
model.anchors.baseline = "Theta = 1-11 \n CONSTRAINB = (5,7,11,a1),
(5,7,11,d)"

# Independent model (completely separate analyses)
m.ind = multipleGroup(dat[paste0("aud___q",1:11)], model.anchors.baseline,
group = groupvar, SE=T, SE.type="SEM", calcNull=T, control=list(maxit=10000),
technical=list(NCYCLES=10000), GenRandomPars = T)

coef(m.ind, IRTpars = T, simplify = T, printSE = T)
M2(m.ind)

# Equal model (slopes and intercepts constrained to be equal)
m.eq  = multipleGroup(dat[paste0("aud___q",1:11)], model.anchors.baseline,
group = groupvar, invariance = c('slopes', 'intercepts'), SE=T,
SE.type="SEM", calcNull=T, control=list(maxit=10000),
technical=list(NCYCLES=10000), GenRandomPars = T)

coef(m.eq, IRTpars = T, simplify = T, printSE = T)
M2(m.eq)

# Freely estimated slopes, intercepts, means, and variances
m.freegroup  = multipleGroup(dat[paste0("aud___q",1:11)],
model.anchors.baseline, group = groupvar, invariance = c('slopes',
'intercepts','free_means','free_var'), SE=T, SE.type="SEM", calcNull=T,
control=list(maxit=10000), technical=list(NCYCLES=10000), GenRandomPars = T)

coef(m.freegroup, IRTpars = T, simplify = T, printSE = T)
anova(m.eq, m.freegroup)
M2(m.freegroup)

# Freely estimated means and variances
m. freeintslope = multipleGroup(dat[paste0("aud___q",1:11)],
model.anchors.baseline, group = groupvar, invariance =
c('free_means','free_var'), SE=T, SE.type="SEM", calcNull=T,
control=list(maxit=10000), technical=list(NCYCLES=10000), GenRandomPars = T)

coef(m.freeintslope, IRTpars = T, simplify = T, printSE = T)
anova(m.eq, m.freeintslope)
M2(m.freeintslope)

# Identify items with DIF using the freegroup model, drop scheme
x.DIF = DIF(m.freegroup, c('a1', 'd'), scheme = 'drop')
n.DIF = which(x.DIF$p < pcrit)
n.DIF = setdiff(n.DIF, c(5,7,11)
vs.DIF = rownames(x.DIF)[n.DIF]
if (length(vs.DIF) == 0){
      print("No DIF detected!")
      return(m.freegroup)
}

DIFs = DIF(m.freegroup, c('a1', 'd'), scheme = 'drop', items2test = n.DIF)
DIFs.a1 = DIF(m.freegroup, c('a1'), scheme = 'drop', items2test = n.DIF)
```

© 2023 Matson TE et al. *JAMA Network Open.*

```
DIFs.d = DIF(m.freegroup, c('d'), scheme = 'drop', items2test = n.DIF)
DIFs.a1.which = rownames(DIFs.a1)[which(DIFs.a1$p < pcrit)]
DIFs.d.which = rownames(DIFs.d)[which(DIFs.d$p < pcrit)]
DIFs.a1.which = as.numeric(gsub("aud___q","",DIFs.a1.which))
DIFs.d.which = as.numeric(gsub("aud___q","",DIFs.d.which))

# Create a final model where anchors + invariant items are constrained to
equality across groups and non-invariant items are free across groups
modeltext.theta = "Theta = 1-11 \n "
modeltext.constrainb = "CONSTRAINB = "

constrain.a1 = paste0(paste((1:11)[-DIFs.a1.which], collapse=","),",a1")
if (length(DIFs.a1.which) == 0) constrain.a1 = paste0(paste((1:11),
collapse=","),",a1")

modeltext.a1 = paste0("(",constrain.a1,"), ")
if (length(DIFs.a1.which) == 11) modeltext.a1 = ""

constrain.d = paste0(paste((1:11)[-DIFs.d.which], collapse=","),",d")
if (length(DIFs.d.which) == 0) constrain.d = paste0(paste((1:11),
collapse=","),",d")

modeltext.d = paste0("(",constrain.d,") ")
if (length(DIFs.d.which) == 0) modeltext.d = ""

modeltext = paste0(modeltext.theta, modeltext.constrainb, modeltext.a1, modeltext.d)
if (length(DIFs.a1.which) == 0 & length(DIFs.d.which) == 0)
modeltext.constrainb = ""

m.final = multipleGroup(dat, paste0("aud___q",1:11)], modeltext, group =
groupvar, invariance = c('free_means', 'free_var'), control=list(maxit=25),
SE=T, SE.type="SEM", calcNull=T)

coef(m.final, IRTpars = T, simplify = T, printSE = T)
anova(m.final, m.ind)
anova(m.final, m.freegroup)
M2(m.freeintslope)

# Basic DTF curve
Plot(m.final, theta_lim=c(-4,4))
```

© 2023 Matson TE et al. *JAMA Network Open.*

eReferences

1. Baker FB. *The Basics of Item Response Theory*. ERIC Clearinghouse on Assessment and Evaluation; 2001.

2. Hallgren KA, Matson TE, Oliver M, et al. Practical Assessment of Alcohol Use Disorder in Routine Primary Care: Performance of an Alcohol Symptom Checklist. *J Gen Intern Med*. Aug 16 2021;doi:10.1007/s11606-021-07038-3

3. Chalmers RP, Counsell A, Flora DB. It Might Not Make a Big DIF: Improved Differential Test Functioning Statistics That Account for Sampling Variability. *Educ Psychol Meas*. Feb 2016;76(1):114-140. doi:10.1177/0013164415584576

4. Lopez Rivas GE, Stark S, Chernyshenko OS. The Effects of Referent Item Parameters on Differential Item Functioning Detection Using the Free Baseline Likelihood Ratio Test. *Applied Psychological Measurement*. 2009/06/01 2008;33(4):251-265. doi:10.1177/0146621608321760

5. Kopf J, Zeileis A, Strobl C. Anchor Selection Strategies for DIF Analysis: Review, Assessment, and New Approaches. *Educ Psychol Meas*. Feb 2015;75(1):22-56. doi:10.1177/0013164414529792

6. Putnick DL, Bornstein MH. Measurement Invariance Conventions and Reporting: The State of the Art and Future Directions for Psychological Research. *Dev Rev*. Sep 2016;41:71-90. doi:10.1016/j.dr.2016.06.004

7. American Psychiatric Association. *Diagnostic and statistical manual of mental disorders (5th ed.)*. 2013.

8. Cheung GW, Rensvold RB. Evaluating Goodness-of-Fit Indexes for Testing Measurement Invariance. *Structural Equation Modeling: A Multidisciplinary Journal*. 2002/04/01 2002;9(2):233-255. doi:10.1207/S15328007SEM0902_5

© 2023 Matson TE et al. *JAMA Network Open.*
