# Supplementary material for: Psychometric Performance of a Substance Use Symptom Checklist to Help Clinicians Assess Substance Use Disorder in Primary Care
Source: JAMA Netw Open. 2023 May 26;6(5):e2316283. doi: 10.1001/jamanetworkopen.2023.16283 (PMC10220521; doi:10.1001/jamanetworkopen.2023.16283)
Supplement: Supplement 2. — Data Sharing Statement [file jamanetwopen-e2316283-s002.pdf]

Data Sharing Statement

Matson. Psychometric Performance of a Substance Use Symptom Checklist to Help Clinicians Assess Substance Use Disorder in Primary Care. *JAMA Netw Open*. Published May 26, 2023. doi:10.1001/jamanetworkopen.2023.16283

Data
Data available: No

Additional Information
Explanation for why data not available: Statistical code is available in the supplement and from Dr. Matson. In keeping with National Institutes of Health values, in which the rights and privacy of individuals must be protected at all times, dataset disclosures to other entities would require (1) a data transfer agreement between Kaiser Permanente Washington and said entities, (2) deidentification of the data set, and (3) appropriate institutional review board approvals. Investigators wishing to obtain these data should contact the corresponding author to discuss the request. Requests may require funding for programming to create a deidentified analytic data set(s) and to establish the appropriate data transfer agreement(s) and institutional review board approval(s).
